# The Molecular Functions of MeCP2 in Rett Syndrome Pathology

**DOI:** 10.3389/fgene.2021.624290

**Published:** 2021-04-23

**Authors:** Osman Sharifi, Dag H. Yasui

**Affiliations:** LaSalle Laboratory, Department of Medical Microbiology and Immunology, UC Davis School of Medicine, Davis, CA, United States

**Keywords:** Rett syndrome, epigenetic, DNA, chromatin, gene expression, MeCP2

## Abstract

MeCP2 protein, encoded by the *MECP2* gene, binds to DNA and affects transcription. Outside of this activity the true range of MeCP2 function is still not entirely clear. As *MECP2* gene mutations cause the neurodevelopmental disorder Rett syndrome in 1 in 10,000 female births, much of what is known about the biologic function of MeCP2 comes from studying human cell culture models and rodent models with *Mecp2* gene mutations. In this review, the full scope of MeCP2 research available in the NIH Pubmed (https://pubmed.ncbi.nlm.nih.gov/) data base to date is considered. While not all original research can be mentioned due to space limitations, the main aspects of MeCP2 and Rett syndrome research are discussed while highlighting the work of individual researchers and research groups. First, the primary functions of MeCP2 relevant to Rett syndrome are summarized and explored. Second, the conflicting evidence and controversies surrounding emerging aspects of MeCP2 biology are examined. Next, the most obvious gaps in MeCP2 research studies are noted. Finally, the most recent discoveries in MeCP2 and Rett syndrome research are explored with a focus on the potential and pitfalls of novel treatments and therapies.

## Introduction

MeCP2 research efforts have been extensive and have therefore greatly advanced the fields of epigenetics, neuroscience and chromatin research. MeCP2, encoded by the *MECP2* gene in humans and the *Mecp2* gene in rodents, was first characterized as a methyl CpG DNA binding protein in 1992, thereby establishing it as an epigenetic reader of DNA methylation (Meehan et al., 1992). It was not until the 1999 discovery that mutations in *MECP2* contribute to the pathology of the rare disease, Rett syndrome (RTT, OMIM #312750), that the relevance of MeCP2 to normal human development was established (Amir et al., 1999). Virtually all Rett patients are female (Reichow et al., 2015) and mosaic for cells with *MECP2* expression as point mutations on the X chromosome are almost always paternally inherited (Trappe et al., 2001). Despite this fact, the function of MeCP2 has been predominately studied in the nervous system of *Mecp2* null male mice as ablation of MeCP2 in all brain cells accounts for the most severe disease phenotypes (Guy et al., 2001). Since the initial characterization of MeCP2 as a DNA binding protein in 1992 (Meehan et al., 1992) a whole field of biochemical research has emerged, culminating in the discovery that MeCP2 can organize chromatin by liquid phase separation (Fan et al., 2020; Li et al., 2020; Wang et al., 2020). Since 1999 and the discovery that mutations in *MECP2* contribute to Rett syndrome, at least 3,194 papers mentioning MeCP2 have accumulated in the NIH Pubmed database. In this review we will examine the biologic function of MeCP2 in mammals, highlight controversial aspects of MeCP2 research, point out significant gaps in knowledge, and report on paradigm shifting advances in the Rett syndrome field.

### Fundamental Aspects of MeCP2 Function in Mammals

#### *MECP2/Mecp2* Mutations and Resulting MeCP2 Deficiencies in Brain Underlie the Majority of Rett Syndrome Like Phenotypes

The key to defining the functions of MeCP2 is to understand the effects that hypomorphic *MECP2* has at the whole organism level. Prior to the identification of *MECP2* mutations in Rett patients, a general model of *MECP2/MeCP2* function had been developed from early studies on the *Mecp2* gene and MeCP2 protein in mice (Lewis et al., 1992). Evidence from *in vitro* studies concluded that MeCP2 acted as a transcriptional repressor of genes in *cis* (Nan et al., 1997). In 1999 a team led by Huda Zoghbi at Baylor College of Medicine and Uta Francke at Stanford University identified the common mutations for Rett syndrome and mapped them to the *MECP2* gene located on the X chromosome (Amir et al., 1999). A Rett syndrome model with a germline *Mecp2* exon 3 and 4 deletion allele produced healthy mice at birth but male pups (*Mecp2^–/y^* null) soon displayed impaired motor skills and premature lethality, while female mice (*Mecp2^–/+^* deficient heterozygotes) became hypoactive and exhibited motor and breathing defects after 3 months of age Post mortem examination of male *Mecp2^–/y^* null mice revealed reduced brain and neuronal size (Guy et al., 2001). These *Mecp2* exon 3 and 4 deletion or “Bird *Mecp2* deletion” mice have been the primary animal model of Rett syndrome research. A simultaneous germline *Mecp2* exon 3 deletion mouse which will be henceforth referred to as the “Jaenisch *Mecp2* deletion” model displayed very similar motor defects and premature death as the Bird *Mecp2* null mice in males and motor, hypoactivity and respiratory phenotypes in females (Chen et al., 2001). To test the hypothesis that *Mecp2* expression is necessary for normal brain function, deletion of *Mecp2* in *nestin* expressing cells, encompassing neurons and glia, also displayed premature death and motor defects in Bird *Mecp2^–/y^* null deletion males and delayed motor defects in Bird *Mecp2^–/+^* deficient heterozygous female mice (Guy et al., 2001). Simultaneous histological examination of Jaenisch *Mecp2^–/y^* null deletion mice revealed reduced neuronal size along with reduced brain weight, suggestive of brain restricted defects underlying Rett like phenotypes (Chen et al., 2001). To better examine the requirement for *Mecp2* expression during brain development, the Jaenisch team also generated mice conditional for *Mecp2* deletion in *nestin* expressing neurons and glia. These mice exhibited the same phenotypes as seen in *Mecp2* germline deletion mice as did mice conditional for *Mecp2* deletion in *CamK* expressing, post-mitotic neurons that presented with delayed neurological defects in both *Mecp2^–/y^* null and *Mecp2^–/+^* deficient female mice and premature death in male mice (Chen et al., 2001). Together, these Bird and Jaenisch germline and conditional *Mecp2* deletion mouse models led to the conclusion that defects in *Mecp2* expression of MeCP2 protein during development is necessary for normal central nervous system function and a normal lifespan. Interestingly, subsequent deletion of *Mecp2* in specific neuronal subtypes revealed specific network defects but not reduced life span (Fyffe et al., 2008; Samaco et al., 2009; Chao et al., 2010) except for male mice with *Mecp2* deletion in somatostatin and parvalbumin neurons (Ito-Ishida et al., 2015). However, *MECP2* exon deletions homologous to *Mecp2* exon deletion model mice are rarely found in Rett patients and thus represent somewhat artificial genetic constructs of the disease.

The requirement for MeCP2 activity during murine development was examined by leveraging a system in which a stop codon in *Mecp2* could be removed using a systemically administered, tamoxifen induced, cre-lox deletion. Using this tool, a landmark series of experiments performed by teams from the Bird and Jaenisch labs indicated that the expression of *Mecp2* in neurons during early adult development was able to rescue motor defects, hypo-activity and premature death in *Mecp2^–/y^* null male mice bearing Bird and Jaenisch *Mecp2* deletion alleles (Giacometti et al., 2007; Guy et al., 2007). To further test the hypothesis that *Mecp2* expression is necessary for neurologic function after development in a whole animal, a team led by Huda Zoghbi examined the effects of the Bird *Mecp2* deletion allele in adult mice. The *Mecp2^–/y^* null male mice acquired motor defects, learning defects, apraxia and lethality within 15 weeks after *Mecp2* gene deletion (McGraw et al., 2011). From these collective *Mecp2* deletion mouse model studies, it is clear that MeCP2 is necessary for normal neuronal function and overall health throughout the lifespan. One caveat of these and other studies is that Bird *Mecp2^–/y^* null deletion males were the primary focus of these studies as they have a robust disease endpoint (death) by 20 weeks of age and present with motor defects at 6 weeks of age (Guy et al., 2001). However, the vast majority of Rett patients are female and are heterozygous for *MECP2* mutations as spontaneous *MECP2* point mutations are passed from the paternal X chromosome in sperm (Trappe et al., 2001). Female *Mecp2^–/+^* deficient heterozygous deletion mice are more difficult to study as life span is normal and the motor, altered anxiety and respiratory symptoms common to *Mecp2^–/y^* null deletion males arise after 6 months of age and are often subtle in presentation (Chen et al., 2001; Guy et al., 2001). It should be noted that maternally inherited *MECP2* mutations are extremely rare, tend to be gene duplications and present as a different disease in male patients (Del Gaudio et al., 2006).

Since the generation of the initial *Mecp2* deletion mouse models, multiple *Mecp2* gene “knock in” models that are based on actual *MECP2* mutations identified in Rett patients have been developed. The model that may have the greatest relevance to Rett is the T158A model developed by Zhou (Goffin et al., 2012) as mutation of threonine 158 is the most common *MECP2* missense mutation according to the RettBASE database (mecp2.chw.edu.au) and affects the ability of MeCP2 to bind to DNA. The non-sense *MECP2* mutation R168X correlates with severe disease symptoms in Rett. This mutation when recapitulated in *Mecp2* resulted in motor, cognitive and anxiety defects in *Mecp2^R168X/y^* males and females, although the time of onset was delayed in females (Schaevitz et al., 2013). In fact, male T158A mice (*Mecp2^T158A/y^*) recapitulated the motor and learning defects as well as premature mortality observed in male *Mecp2* null mice (Goffin et al., 2012) while female *Mecp2^T158A/+^* exhibit breathing abnormalities similar to those observed in Rett females (Bissonnette et al., 2014). In 2014 a mouse model based on a human *MECP2* exon 1 mutation showed motor defects similar to Bird *Mecp2* null mice along with altered anxiety and stereotypic behavior in *Mecp2-e1^–/y^* males (Yasui et al., 2014). Additional *Mecp2* knock in mouse models based on Rett *MECP2* mutations have been developed and exhibit a range of motor and behavioral phenotypes as reviewed in Schmidt and Cardoso (Schmidt et al., 2020). Rat *Mecp2* knock in models may have advantages over mouse models as a *Mecp2* truncation model exhibits neurologic regression in *Mecp2^–/+^* females and thus more accurately models Rett syndrome than some existing mouse models (Veeraragavan et al., 2015).

#### Mutations in *MECP2/Mecp2* Affect Virtually All Organs and Tissues

**FIGURE 1:**
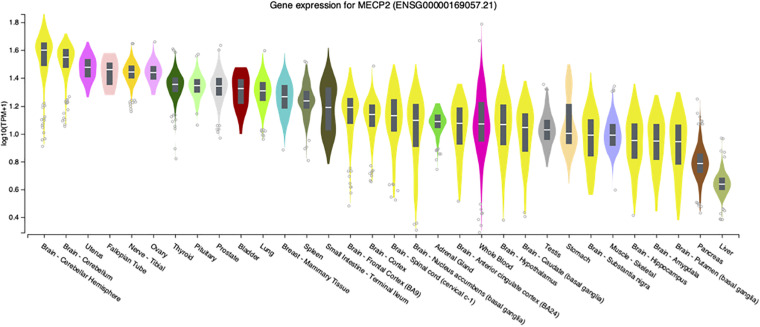
*MECP2* transcript levels in select human tissues. Violin plots from the Genome-Tissue expression (GTEx) project are shown for *MECP2* with log2 values ranked from high to low levels.

Although the most severe disease phenotypes were observed in *Mecp2*^–/y^ null deletion male mice with deletion engineered in brain (Chen et al., 2001; Guy et al., 2001), defects in *MECP2* expression in humans has the potential to affect almost all organs and cell types, as transcripts are detected at significant levels in virtually all adult human tissues (Figure 1). At the systems biology level, male mice engineered to have only peripheral deletion of *Mecp2* outside of the nervous system were hypoactive, had reduced exercise capacity and bone defects, but survived beyond 50 weeks after birth (Ross et al., 2016). This study, led by Stuart Cobb, established that *Mecp2* expression in multiple organs and cell types likely accounts for these broad phenotypes thereby underlining the importance of MeCP2 function outside of the nervous system.

These limited studies indicate that while MeCP2 is necessary for normal neurologic function in mice, disease is still present in other tissues. Similarly, loss or diminished MeCP2 activity contributes to Rett syndrome pathology outside of the nervous system. For example, an early report in 1994 described extended QTc intervals in Rett patients (Sekul et al., 1994). Analysis of *Mecp2* deletion mice revealed similar cardiac abnormalities as the patients as well as ventricular tachycardia (McCauley et al., 2011). To further investigate this finding, conditional deletion of *Mecp2* from cholinergic neurons was performed by a team led by Jeff Neul. They found that loss of *Mecp2* from cholinergic, parasympathetic neurons recapitulated the previous cardiac findings in Bird *Mecp2* null mice and symptoms in Rett patients (Herrera et al., 2016). Interestingly, while *MECP2* transcripts have relatively low abundance in the human liver (Figure 1) an ENU mutagenesis screen in Bird *Mecp2^–/y^* null male mice revealed that MeCP2 function is required for normal lipid metabolism as these animals have dysregulated cholesterol synthesis in brain and elevated cholesterol in liver and serum (Buchovecky et al., 2013; Kyle et al., 2016; Kyle et al., 2018). *MECP2* is also expressed at high level in human intestine (Figure 1) and 66% of Rett patients report gastrointestinal pain (Symons et al., 2013). A 2016 study found that *Mecp2* null mice had gut hypomotility and reduced nitric oxide synthase expression in enteric neurons (Wahba et al., 2015). To examine the hypothesis that microbial alterations underlie intestinal defects, the overall gut microbiome diversity was examined and appeared to be reduced in Rett patients (Strati et al., 2016; Borghi et al., 2017). As the diets were similar between the Rett and control subjects, these results suggest that altered *MECP2* levels in the digestive tract may alter the intestinal environment and thus microbe growth. In fact, it was recently shown that mice with loss of *Mecp2* expression solely in the intestine have severe colonic epithelial defects (Millar-Büchner et al., 2016). As there is direct signaling from the gut to the brain, extension of these gut/microbiome studies should be extended to Bird *Mecp2*^–^*^/+^* deficient female deletion mice and would thus provide critical insights for female Rett patients. While studies on the role of MeCP2 function in tissues outside of the brain, a recent study found evidence that MeCP2 represses LINE-1 activity in brain, but not in peripheral tissues such as heart and eye (Zhao et al., 2019).

#### MeCP2 Is a DNA Binding Protein

MeCP2 was first described in the lab of Adrian Bird as a DNA binding protein with a high affinity for CpG methylated DNA (Meehan et al., 1992) via the methyl DNA binding domain (MBD) (Free et al., 2001). Although this report is often overlooked, MeCP2 was later found to be homologous to the nuclear matrix binding protein ARBP, cloned from chicken (Weitzel et al., 1997). This finding is intriguing as the nuclear matrix structures the nucleus in eukaryotic cells and organizes chromatin for replication, DNA repair and transcription among other functions (Wasag and Lenartowski, 2016). These *in vitro* binding results from the Stratling lab suggested that ARBP/MeCP2 was involved in chromatin loop organization and heterochromatin structure (Weitzel et al., 1997). Basic biochemical analyses of MeCP2 by Jeff Hansen and colleagues revealed that MeCP2 is a highly disordered protein outside of the MBD domain (Adams et al., 2007). Significantly, another study by the Hansen and Woodcock team demonstrated that a common Rett *MECP2* mutation, R106W in the MBD altered MeCP2 interaction with nucleosomal DNA (Nikitina et al., 2007). Consistent with these results, MeCP2 lacking the MBD domain was found to bind with low affinity to chromatin *in vivo* (Stuss et al., 2013). The Hansen group later determined that MeCP2 contains at least two distinct DNA binding domains, the MBD and sequences in the carboxyl terminus (Ghosh et al., 2010).

While Woodcock and colleagues had identified DNA binding regions in MeCP2 outside of the MBD, these sites were not well characterized at the time (Ghosh et al., 2010). Later however, these non-MBD binding sites were identified as AT hook regions by the Zoghbi lab in 2013 (Baker et al., 2013). The description of AT hook domains in MeCP2 fits well with an 2005 description of high affinity binding sites containing AT runs adjacent to methylated CpG sites (Klose et al., 2005). To summarize these diverse findings, a very recent paper nicely illustrates the *in vivo* activity of MeCP2 in live cells. In this study Nat Heintz and colleagues leveraged MeCP2 fluorescent tagging and real-time visual mobility analysis to describe the sum effect of stable MBD domain interactions and transient AT hook interactions that slow the diffusion of MeCP2 from DNA in living cells (Piccolo et al., 2019).

Since the early characterization of MeCP2 as a methylated CpG binding protein (Meehan et al., 1992), the range of MeCP2 binding activity has been extended. A discovery by the Heintz lab revealed that 5-hydroxymethylcytosine (5hmc) is highly abundant in mouse brain (Kriaucionis and Heintz, 2009). This finding was confirmed by Anjana Rao and colleagues who also identified TET1 as the enzyme responsible for the conversion of 5-methylcytosine (5mc) to 5hmc in brain (Tahiliani et al., 2009). Later it was determined by the Heintz lab that MeCP2 can bind to 5hmc with similar affinity as 5mc in actively transcribed genes (Mellén et al., 2012). However, *in vitro* results had previously shown that 5hmc inhibits binding of the MBD domain of MeCP2 to DNA (Valinluck et al., 2004). Although the activity of TET1 and related factors TET2 and TET3 can each lead to active CpG demethylation, potentially altering the transcriptional state of genes (He et al., 2011) the biologic implications of MeCP2 binding to 5hmc is still not clear.

While 5mc and 5hmc in CpG dinucleotides account for the majority of MeCP2 binding sites in the mammalian genome, MeCP2 can bind to other motifs. In 2014 the Hongjun Song lab revealed that surprisingly, 25% of CpA, CpC, and CpT (CH) dinucleotides are methylated in the brain compared with 75% of CpG sites (Guo et al., 2014). The Song lab also showed that MeCP2 was able to bind to methylated CpH *in vitro* (Guo et al., 2014). The following year, Harrison Gabel and other members of the Greenberg lab determined that MeCP2 binds to methyl CpA sites within gene bodies, although the effect of this binding is controversial as described in a subsequent section of this review (Gabel et al., 2015). More recently the binding of MeCP2 was correlated with the methylation status of CH sites in the brain (Lavery et al., 2020). These results conflict with data indicating that MeCP2 binding to DNA is mostly due to CpG methylation and that MeCP2 binds to promoters with low CpG density (Baubec et al., 2013). While it is clear that MeCP2 binds to dinucleotides with methylated cytosine, an unbiased statistical analysis of genome-wide MeCP2 ChIP-seq data sets indicated that the GC content of a particular genomic region is the most predictive of MeCP2 binding (Rube et al., 2016). In contrast to these results, recent *in vitro* and *in vivo* studies indicate that MeCP2 has minimal binding to non-methylated GT rich DNA (Connelly et al., 2020). To further define MeCP2 binding, Buchmuller et al. (2020) found that MeCP2 was able to bind to DNA *in vitro* with the asymmetric cytosine modifications C/mC, mC/mC, mC/hmC, and mC/fC, where C is cytosine, mC is 5 methyl cytosine, hmC is 5 hydroxymethylcytosine and fC is 5 formylcytosine. These recent findings appear to account for the ability of MeCP2 to bind virtually anywhere in the genome. It is important to note that the level of MeCP2 protein expression is critical for normal brain function as mice with 50% of normal expression (Kerr et al., 2008; Samaco et al., 2008) and twice normal expression (Collins et al., 2004) exhibit neurological defects. Recent studies suggest that altered neuronal MeCP2 levels correlate with heterochromatin changes and behavioral symptoms (Ito-Ishida et al., 2020).

#### MeCP2 Is Involved in Higher Order Chromatin Organization

Another fundamental aspect of MeCP2 function that is often overlooked is its ability to regulate large chromosomal domains. For example, some of the earliest *in vitro* structural studies revealed that MeCP2 could bind and condense nucleosomal arrays that incorporate both methylated and unmethylated DNA (Georgel et al., 2003). Interestingly, these studies did not indicate that MeCP2 was able to form dimers, suggesting that one MeCP2 molecule could bridge two independent arrays. A similar chromatin condensation activity was shown in myoblast cell lines by the Cardoso lab (Brero et al., 2005). Co-immunoprecipitation studies by the El-Osta lab found that the SWI/SNF chromatin remodeling factors Brahma and BAF57 can be recruited to DNA by MeCP2 (Harikrishnan et al., 2005). While these studies examined recombinant MeCP2 linking of artificial nucleosomal constructs, a study from the Kohwi-Shigematsu lab identified a functional chromatin looping activity *in vivo*. In this study Shin-Ichi Horike and colleagues found that MeCP2 mediated long range looping and thus transcriptional activity of the *Dlx5* gene (Horike et al., 2005). In a similar study it was found that long range interactions between the Prader-Willi imprinting center (PWS-IC) and *CHRNA7* modulated gene expression in neurons (Yasui et al., 2011). These studies in cell lines were also consistent with *in vitro* studies from the Hansen lab revealing that MeCP2 was able to interact with DNA outside of the MBD domain, thereby accounting for the ability of one MeCP2 molecule to bind to two sites simultaneously in chromatin looping (Nikitina et al., 2007). This 2007 study also found that MeCP2 molecules encoded by frequent Rett *MECP2* mutations were deficient in their ability to compact chromatin (Nikitina et al., 2007). Studies from the LaSalle lab extended this chromatin looping aspect of MeCP2 function further by showing that MeCP2 along with CTCF contributes to both inter and intra chromosomal interactions and gene regulation of 15q11-13 (Meguro-Horike et al., 2011; Yasui et al., 2011). Later studies from the Natalie Berube lab showed that MeCP2 recruits ATRX to imprinted genes to regulate CTCF chromatin looping interactions (Kernohan et al., 2014). Recent atomic force microscopy (AFM) studies reveal that MeCP2 forms loops by bridging distant binding sites on continuous DNA strands (Liu et al., 2020).

#### MeCP2 Regulates RNA Splicing

While MeCP2 can affect gene transcription directly, one relatively unexplored activity of MeCP2 is its effect on RNA splicing. The first genome wide study of MeCP2 effects on splicing was performed in the Huda Zoghbi lab in 2005. These studies revealed significant splicing defects in *Mecp2^308/y^* male mice expressing a truncated MeCP2 protein (Young et al., 2005). *In vitro* studies revealed that MeCP2 bound to RNA along with YB-1 resulting in gene construct splicing events in neuronal cell lines (Young et al., 2005). Following this report, few attempts were made to confirm the role of MeCP2 in RNA splicing regulation. It was not until 2013 that the Keji Zhao lab reported that *Mecp2* ablation correlated with alternatively spliced exon skipping (Maunakea et al., 2013). Research from the LaSalle lab in 2014 confirmed MeCP2 association with YB-1 shown by Young et al. (2005) as well as interaction with the RNA splicing factors MATR3, SFPQ, and SFRS1 (Yasui et al., 2014). The mechanism underlying MeCP2 regulation of splicing was further explored by the Rasko lab. They found that MeCP2 depletion reduces splicing factor recruitment to methylated DNA and increases intron retention events in blood cells (Wong et al., 2017). More recently Nurit Ballas and colleagues reported abnormal intron retention exon skipping in activation induced genes in *Mecp2* null neurons compared to wild-type neurons (Osenberg et al., 2018). The latest investigation of the role of MeCP2 in RNA splicing regulation using a machine learning approach, however, found subtle changes in RNA splicing in cells with varying levels of MeCP2 (Chhatbar et al., 2020). While the results may be not always be direct, or of great magnitude, MeCP2 binding to DNA has an effect on mRNA splicing events. However, one wonders how much current RNA-seq methodologies bias this sort of approach.

### Unresolved Details of MeCP2 Function

#### MeCP2 Functions as a Transcriptional Repressor and Activator

The model of MeCP2 as a direct transcriptional repressor of single copy genes in *cis* derived from *in vitro* studies dating back to 1992. In 1997 this model was formally proposed in a report showing that affinity purified rat MeCP2 showed repressive activity of CpG methylated gene promoter constructs *in vitro* (Nan et al., 1997). Subsequent studies from the Alan Wolffe lab demonstrated that MeCP2 recruitment of histone deacetylase activity was correlated with transcriptional repression (Jones et al., 1998). An example of MeCP2 functioning as a transcriptional repressor is the regulation of viral elements that exist in the genome as multiple, highly CpG methylated elements. Specifically, LINE-1 elements were found to be transcriptionally repressed by MeCP2 binding (Lorincz et al., 2001; Yu et al., 2001). In 2010, a paradigm shifting report published by the Gage lab found that mammalian neuronal progenitor cells have a massive activation of LINE-1 transcription and transposition that is absent in cells from peripheral tissues (Muotri et al., 2010). More recently the Cardoso lab demonstrated in cell lines that MBD proteins including MeCP2 repress TET1 mediated 5hmc conversion and activation of LINE1 elements in human cells (Zhang et al., 2017). A recent analysis of LINE1 insertions revealed in that Rett tissues had fewer cells with exonic insertion, consistent with selection against neurons with multiple LINE1 insertion events and other cell types (Zhao et al., 2019). Thus, *in vitro* and *in vivo* evidence indicates that MeCP2 binds to CpG methylated DNA promoters and represses transcription of genes and viral elements along with co-factors such as HDACs.

The model of MeCP2 as a transcriptional repressor of single copy genes was first challenged by the genome wide correlation of MeCP2 binding with gene transcripts. In 2007, MeCP2 ChIP-seq analysis combined with gene transcript analysis from the LaSalle lab found that MeCP2 bound to active promoters with low levels of DNA methylation (Yasui et al., 2007). This was closely followed by results published in 2008 from the Zoghbi lab showing that loss of MeCP2 correlated with both reduced and elevated levels of gene transcripts in murine hypothalamus (Chahrour et al., 2008). Further analyses of select gene promoters upregulated by loss of MeCP2 activity revealed binding of the transcriptional activator CREB1 along with MeCP2 to expressed genes (Chahrour et al., 2008). In support of these findings, in 2017 it was shown that *MECP2* mutant and *MECP2* null human embryonic stem cell derived forebrain neurons had reduced CREB levels along with reduced dendritic complexity, neurite growth, and mitochondrial function (Bu et al., 2017). Most recently, results from the Harrison Gabel lab suggest that DNA methylation at enhancers may repress genes in trans (Clemens et al., 2020). Clearly these results show that MeCP2 binding and recruitment of co-factors correlates with both activation and repression of transcription. However, the location of MeCP2 binding and co-factor interactions appear to be critical for how gene expression is affected.

#### MeCP2e1 and MeCP2e2 Protein Isoforms Have Distinct Functions

**FIGURE 2:**
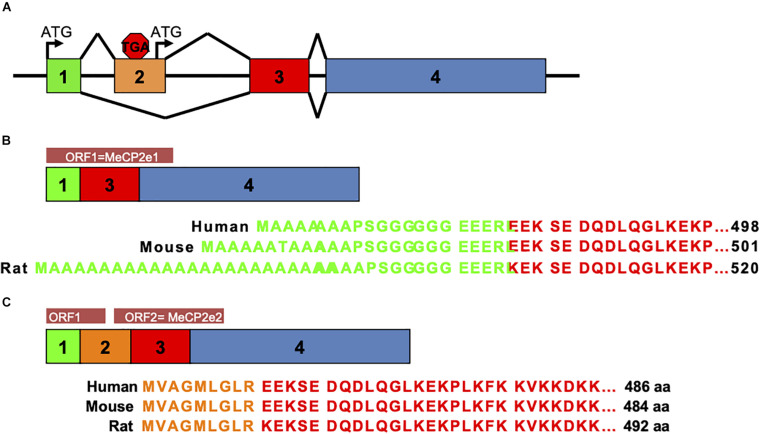
MeCP2 protein isoforms. MeCP2e1 and MeCP2e2 protein isoforms are produced from alternative splicing events. The MeCP2e1 isoform is translated from splicing of exon 1 to exons 3 and 4 with the translational start ATG in exon 1. The MeCP2e2 protein isoform is produced from a transcript from all four exons with a translational start ATG in exon 2. Interestingly, this transcript may encode a small peptide from an ORF in exons 1 and 2. **(A)** *MECP2/Mecp2* exon splicing. **(B)** MeCP2-e1 isoform. **(C)** MeCP2-e2 isoform.

Prior to 2004, MeCP2 was believed to be the gene product of three exons comprising one protein isoform. In that year a team from The Hospital for Sick Children in Toronto described a novel exon upstream of the known *MECP2* exons that could splice to exons 3 and 4 to produce a novel protein isoform with a distinctly different amino terminus than the originally described MeCP2 isoform (Mnatzakanian et al., 2004). The presence of this novel exon was also shown in mouse *Mecp2* by RT-PCR (Mnatzakanian et al., 2004). Around the same time, the homologous upstream exon in murine *Mecp2* was also reported by the lab of Adrian Bird (Kriaucionis and Bird, 2004). Therefore, the alternative splicing of *MECP2/Mecp2* exon 1 to exons 3 and 4 produces the MeCP2e1 isoform while translation of mRNA with all four exons leads to production of MeCP2e2 due to the use of an alternative translational start site (Figure 2). In humans and rodents this produces MeCP2 isoforms which have almost identical amino acid sequences but with distinctly different amino termini (Figure 2). Furthermore, it was shown that mutations in *MECP2* exon 1 are present in Rett patients (Mnatzakanian et al., 2004) and that *MECP2* exon 1 mutations disrupt translation of the MeCP2e1 but not MeCP2e2 isoforms (Gianakopoulos et al., 2012).

Evidence that MeCP2e1 is the isoform underlying Rett syndrome was provided by two key studies. In the first study, knockout of *Mecp2* exon 2 which only effects production of MeCP2e2 resulted in mice with normal development and function (Itoh et al., 2012). However, *Mecp2* exon 2 deletion did result in placental defects (Itoh et al., 2012). To follow up on these findings the Berge Minassian lab screened a cohort of patients with idiopathic RTT. In one patient they found an *MECP2* exon 1 A to T point mutation affecting the translational start site (Saunders et al., 2009). This exon 1 mutation was predicted to prevent translation of only the MeCP2e1 isoform but not the MeCP2e2 isoform, suggesting that it is the loss of MeCP2e1 activity that results in Rett syndrome. Therefore, the LaSalle lab replicated this mutation in a mouse model which resulted in the complete loss of the MeCP2e1 protein isoform while retaining expression of MeCP2e2 (Yasui et al., 2014). Most importantly the results from Yasui and colleagues found that *Mecp2e1^–/y^* males had impaired motor function, exhibited apraxia like limb clasping with altered anxiety behavior and premature death (Yasui et al., 2014) similar to Bird *Mecp2* deletion male mice (Guy et al., 2001). These MeCP2e1 deficient mice accurately model Rett syndrome as heterozygous *Mecp2e1^–/+^* females exhibited significant motor impairment and altered body composition (Yasui et al., 2014; Vogel Ciernia et al., 2017). Interestingly, both *Mecp2e1^–/y^* males and *Mecp2e1^–/+^* females had elevated body fat accumulation correlating with reduced energy expenditure in early adulthood (Vogel Ciernia et al., 2018).

Additional evidence that MeCP2e1 has non-overlapping function with MeCP2e2 is the finding by the Rastegar lab that MeCP2e1 is expressed in brain prior to MeCPe2 at higher levels and a more consistent distribution pattern between brain regions (Olson et al., 2014). Consistent with the mouse studies, the Ellis lab found that human MeCP2e1 deficient iPS derived neurons recapitulate synaptic current, soma size, membrane potential and maturation defects common to Rett syndrome further establishing the necessity of MeCP2e1 in neuronal development (Djuric et al., 2015). MeCP2e1 and MeCPe2 were also found by the Ausio lab to have differential DNA binding kinetics, distinct co-factor association and preferred different DNA binding motifs (Martínez De Paz et al., 2019). Perhaps the most compelling evidence that MeCP2e1 has an essential function that differs from MeCP2e2 is the finding that MeCP2e1 is the evolutionarily older isoform, as orthologs have been identified in vertebrates back to bony fish (Osteichthyes) and amphibians (Mnatzakanian et al., 2004).

#### Glial Cells Are Critical for Rett Syndrome Pathology

Another contentious issue in the MeCP2/Rett field is the contribution of non-neuronal cells to the disease process, despite considerable evidence that *MECP2* transcripts are present at high levels in virtually all tissues (Figure 1). Although cell sorting experiments show that MeCP2 is present at near octomer levels in neurons, MeCP2 is present in glia, albeit at much lower levels (Skene et al., 2010). Direct evidence for the role of non-neural cells in Rett pathology was provided by research performed by the Jin lab at UC Davis where it was found that astrocytes from Bird *Mecp2^–/y^* null male mice were abnormal and produced underdeveloped wild-type neurons compared with wild-type astrocytes in a co-culture system (Maezawa et al., 2009). A subsequent study by the Jin lab revealed that Bird *Mecp2^–/y^* null male microglia damaged adjacent neurons in a non-cell autonomous manner by releasing glutamate (Maezawa and Jin, 2010). The relevance of cell autonomous vs. non-cell autonomous effects in the *Mecp2* mutant brain is discussed further in the next section.

In support of these studies, a paper by the Mandel lab described that re-expression of *Mecp2* solely in astrocytes was able to reduce motor defects, breathing irregularity as well as prolong life in Bird *Mecp2^–/y^* null male mice (Lioy et al., 2011). Surprisingly, microglia, which comprise a tiny percentage of brain cells, were found to be important for Rett pathology when it was shown that replacement of *Mecp2* null microglia with wild-type microglia in Bird *Mecp2^–/y^* null male brain improved life span, normalized breathing and reduced motor impairment (Derecki et al., 2012). Although these results were not independently reproduced, a recent study by the Stevens lab established that microglial engulfment of synapses damages neural circuits in *Mecp2^–/y^* null male mice (Schafer et al., 2016). To further address the specific contribution of astrocytes to Rett phenotypes, Qiang Chang from the University of Wisconsin Madison derived wild-type and *MECP2* mutant iPS astrocytes and examined their function in culture with neurons. They found that *MECP2* mutant astrocytes have excess calcium stores that correlated with elevated NMDA receptor expression in neighboring neurons and hyper neuronal network excitability (Dong et al., 2018). Therefore, the authors conclude that astrocytes act both cell-autonomously and non-cell autonomously to mediate Rett like pathology (Dong et al., 2018). An extensive review on the function of MeCP2 in glia has recently become available (Kahanovitch et al., 2019).

#### *MECP2/Mecp2* Has Both Cell Autonomous and Non-cell Autonomous Effects

The vast majority of Rett patients are female *MECP2* heterozygotes and are thus cellular mosaics for wild-type and mutant *MECP2* expressing cells. It was first reported by the LaSalle lab that Bird *Mecp2* null neurons affected the expression of adjacent wild-type neurons in *Mecp2^–/+^* heterozygous female brains (Braunschweig et al., 2004). This finding led to the “bad neighborhood” or non-cell autonomous effects hypothesis of *Mecp2* deficiency where defects in *Mecp2* mutant cells affect the function of local wild-type cells in the brain or other tissues (Braunschweig et al., 2004). This “bad neighborhood” becomes important when examining disease processes in female but not male mice where all cells are deficient in *Mecp2* expression. Subsequent studies by the Gail Mandel lab showed that MeCP2 deficient glia impair neuronal function in a non-cell autonomous manner *in vitro* (Ballas et al., 2009). As mentioned in the previous section, a similar *in vitro* culture system showed that *Mecp2^–/y^* null astrocytes were able to impair the growth of wild-type neuronal dendrites through soluble factors (Maezawa et al., 2009). To study these effects *in vivo* by transplanting either *Mecp2* mutant or wild-type neuroblasts into wild-type brain Kishi and Macklis found that MeCP2 functions largely cell autonomously in development of the cortex but that non-cell autonomous effects on neuronal function exist (Kishi and Macklis, 2010). In the most definitive study to date, the Zhou lab examined *Mecp2* mutant tagged and wild-type cells from female brains and found that non-cell autonomous effects were greater in magnitude than cell autonomous effects and tended to effect cell to cell signaling and phosphorylation (Johnson et al., 2017). These results were consistent with subsequent single cell RNA-sequencing (sc-RNAseq) analysis of neurons from *Mecp2*^–/+^ deficient female brain suggesting that MeCP2 can affect gene expression in a non-cell autonomous manner in different neuronal cell types, Renthal et al. (2018).

#### MeCP2 Preferentially Regulates Long Genes Over Short Genes

The hypothesis that MeCP2 preferentially regulates long genes was first proposed by Sacha Nelson in 2014 (Sugino et al., 2014). This report was supported by data published from the Greenberg lab in 2015 (Gabel et al., 2015). This hypothesis was further tested in an important study mentioned previously by the Zhou lab which found a trend toward long genes being more severely affected by MeCP2 deficiency than short genes (Johnson et al., 2017). However, these findings were refuted by a re-analysis of the data and transcriptional analysis using alternative methods which together, suggested that traditional PCR methods bias the RNA-seq results toward long genes (Raman et al., 2018). The controversy over regulation of long genes is discussed further in a review by Connolly and Zhou (2019).

#### At What Developmental Stage Do MeCP2 Defects Impair Development

A lack of a developmental time course of how MeCP2 defects are manifested impedes Rett research. Although there is an abundance of data about MeCP2 defects in adult mice, the earliest stages of development have not been well characterized, aside from a 2003 study which found MeCP2 positive cells in E14 rat cortex (Jung et al., 2003). To address this gap, the Landsberger lab analyzed neurons from embryonic Bird *Mecp2^–/y^* null male cortex (Bedogni et al., 2016). They found that Bird *Mecp2^–/y^* null embryonic neurons have altered gene expression, altered morphology, reduced calcium flux and mobility compared to wild-type neurons (Bedogni et al., 2016). Independent evidence suggests that there are deficiencies in *Mecp2* transcripts and MeCP2 protein in the mouse cortex as early as E14 (Zachariah et al., 2012). However, one limitation of these molecular studies of early MeCP2 expression in mice is that they employed conventional methods in bulk tissue such as qRT-PCR and Western blot and that the investigators studied Bird *Mecp2^–/y^* null male mice which do accurately model Rett syndrome in *MECP2* heterozygous females. To date there has been only one study of MeCP2 embryonic function at single-cell resolution in disease relevant Bird *Mecp2^–/+^* deficient mouse brain by the Greenberg group. However only adult Bird *Mecp2^–/+^* deficient deletion female mice and adult human Rett brains were analyzed (Renthal et al., 2018). Yet it is known that Rett syndrome girls experience a postnatal developmental regression at about 6–18 months of age (Zoghbi, 2005). Thus, the molecular changes during this critical early time are still not well defined. While it is clear that MeCP2 function is critical for normal neurological function, the molecular phenotypes in distinct brain cell types have not been investigated over the full-time course of development in mouse models. The Rett field is lacking an extensive, longitudinal study in mosaic *Mecp2^–/+^* deficient mice investigating the mechanisms and molecular pathways that are impacted by perturbed *Mecp2*/MeCP2 expression throughout disease progression.

#### MeCP2 Is an RNA Binding Protein

Coding RNAs and non-coding RNA such as long non-coding RNAs, small nucleolar RNAs, and micro RNAs establish a diverse set of functions due to their direct interactions with RNA-binding proteins (RBPs). In one of the few studies on this topic it was found that MeCP2 binds with high affinity to mRNA and siRNA outside of the MBD *in vitro* (Jeffery and Nakielny, 2004). MeCP2 was also found to associate with long non-coding RNAs and imprinted genes in mouse brain extracts (Maxwell et al., 2013). To examine MeCP2 interaction with RNA *in vivo*, MeCP2 RNA-immunoprecipitation of small RNAs was performed in the lab of Assam El-Osta who found that MeCP2 bound to specific RNAs including micro RNAs (Khan et al., 2017). These studies investigating MeCP2 interactions with RNA were performed in a targeted manner, therefore an unbiased, genome wide screen of RNA binding would provide a significant advance in understanding this MeCP2 activity.

### Recent Developments in the MeCP2 Field

#### MeCP2 Enables Liquid Phase Separation Events in Nuclear Compartmentalization

In the last 2 years the biologic functions of MeCP2 have expanded appreciably. One concept that has emerged is the effect that liquid-liquid phase separations (LLPS) have on the formation of sub-nuclear compartments (Alberti et al., 2019). In their review Alberti, Gladfelter, and Mittag explain that the nucleus of eukaryotic cells consists of membrane less structures such as nucleoli and heterochromatin that form by LLPS through the activity of proteins and nucleic acids that condense into a dense phase (heterochromatin) and a loose phase depending on conditions such as molecular concentration, salt concentration and pH (Alberti et al., 2019). Thus, the three near simultaneous reports that MeCP2 is involved with LLPS DNA phase separation in the cell nucleus was of great relevance to the field. In the first report, Wang and colleagues found that MeCP2 was able to condense chromatin constructs *in vitro* and that DNA methylation enhanced this effect (Wang et al., 2020). Furthermore, Wang et al found that mutant MeCP2 proteins from Rett patients were defective in chromatin condensation and LLPS (Wang et al., 2020). Results from Fan and colleagues confirmed that wild-type MeCP2 was able to form condensates with DNA *in vitro* (Fan et al., 2020). Finally, research published by Richard Young and Rudolph Jaenisch at MIT also showed that MeCP2 condensed chromatin *in vitro* and MeCP2 mutant forms were defective in LLPS activity (Li et al., 2020). What set apart the Young and Jaenisch study from the other studies is the finding that the intrinsically disordered regions (IDR) of MeCP2 mediate the phase separation activity in the nucleus (Li et al., 2020). The concept of MeCP2 as a nuclear organizer via LLPS is reminiscent of early reports that an MeCP2 homolog, ARBP, functions as a component of the nuclear matrix as mentioned previously (Weitzel et al., 1997).

#### MeCP2 Functions With DNMT3A/Dnmt3a to Regulate Gene Expression

MeCP2 and DNMT3A, a *de novo* DNA methyltransferase have long been linked by the fact that the MeCP2 MBD preferentially binds to CpG methylated DNA (mCG). Recently it was found that there is a direct molecular link between the two molecules as the MeCP2 TRD binds to the DNMT3A ADD domain. This interaction inhibits DNMT3A activity and this inhibition is countered by DNMT3A interaction with H3K4 through the ADD domain *in vitro* (Rajavelu et al., 2018). Similar results were shown by a collaboration between the Joe Ecker and Huda Zoghbi labs that examined the *in vivo* effects of MeCP2 and DNMT3A interaction. It had been previously established by the Ecker lab that high levels of DNA methylation at CH (mCH) sites (H = Adenosine, Cytosine, and Thymidine) in neurons, is due to the activity of DNMT3A (Lister et al., 2009). Therefore, Zoghbi and Ecker examined the hypothesis that as MeCP2 is the sole reader of methyl mCH and that DNMT3A is the sole writer of methyl CH, deletion of either factor in neurons would have similar effects on gene transcription. Surprisingly, loss of DNMT3A in neurons affected transcription of significantly more genes than the loss of MeCP2, highlighting the importance of mCH to neurologic gene regulation and function (Lavery et al., 2020). The relationship between mCH, mCG, and MeCP2 during development is explored further in a review by Lavery and Zoghbi (2019). These advances also underscore the concept that much of MeCP2 function is mediated through co-factor association.

#### MeCP2 May Function in DNA Repair Processes

The first evidence to suggest that MeCP2 could function in DNA repair processes was first reported in 2005. Valinluck and colleagues found that MeCP2 binds with high affinity to DNA containing halogenated pyrimidines, a modification that can result from inflammation (Valinluck et al., 2005). More recently it was found that neural stem cells from Bird *Mecp2*^–/+^ deficient female mice are prone to early senescence and have elevated rates of cell death when exposed to DNA damaging H_2_O_2_, UV light and doxorubicin (Alessio et al., 2018). However, the most convincing evidence for the role of MeCP2 in DNA repair comes from an unbiased N-ethyl-N-nitrosourea (ENU) suppressor screen in Bird *Mecp2^–/y^* null male mice (Enikanolaiye et al., 2020). Out of 2,498 males born to ENU treated wild-type males mated with *Mecp2^–/+^* females, 96 males had ameliorated neurologic symptoms from which 32 genes which suppressed the *Mecp2* null phenotype were identified. Of these 32 genes, *Tet1*, *Birc6*, and *Spin1* function in the DNA damage response while *Rbbp8, Rad50, Fan1, Brca1*, and *Brca2* encode factors involved in DNA double strand break repair (Enikanolaiye et al., 2020).

#### Potential Treatments Under Development for Rett Syndrome

The remarkable reversal of Rett like phenotypes in Bird *Mecp2^–/y^* null male mice provides a basis for clinical therapies that seek to restore expression of *MECP2* (Guy et al., 2007). To this aim, the potential of adeno associated viral (AAV) vectors to deliver MeCP2 to deficient cells in a whole animal model was shown independently in 2013 by two groups. One group led by Stuart Cobb showed that AAV9/*MECP2* delivered intravenously in pre-symptomatic Bird *Mecp2^–/y^* null male mice extended survival modestly (Gadalla et al., 2013). Gail Mandel led a second study that showed that intravenous AAV9/MeCP2e1 vector reduced motor defects in Bird *Mecp2^–/+^* deficient female mice, which thus had greater relevance to Rett syndrome as 95% of Rett patients are female (Garg et al., 2013). In 2017, after these early proofs of principle, advances in *AAV9/MECP2* gene therapy were reported by the combined efforts of Stuart Cobb and Steven J. Gray. The authors in two manuscripts reported improvement in cell transduction with a reduced titer of a second generation AAV9/hMeCP2 virus, a reduction in liver toxicity and prolonged survival in Bird *Mecp2^–/y^* null mice by delivery through the cerebral spinal fluid (CSF) or directly into brain (Gadalla et al., 2017; Sinnett et al., 2017). These studies, while they represent significant improvements in *MECP2* gene therapy tools, should be performed in Bird *Mecp2^–/+^* deficient heterozygous female deletion mice to be more relevant to Rett. Furthermore, the risks of treating human children with AAV vectors cannot be fully mitigated.

Alternatively, the use of site directed RNA editing is being developed for potential Rett syndrome therapy. RNA editing strategies leverage an Adenosine Deaminase Acting on RNA (ADAR2) RNA editing factors with a guide RNA to repair *Mecp2/MECP2* point mutations in RNA. The first report on *Mecp2* RNA editing was published by the Gail Mandel lab in 2017 where an exogenous, modified ADAR2 and guide was used to successfully edit 72% G to A mutations in RNA from *Mecp^R106Q^* mouse neurons (Sinnamon et al., 2017). In 2020 Sinnamon and colleagues successfully edited up to 50% of mRNA in neurons in developing brains of *Mecp2* mutant mice using a similar approach (Sinnamon et al., 2020). The potential of RNA editing as a Rett therapy is limited by the fact that although endogenous RNA editing proteins such as ADAR1, exist naturally in the brain, current approaches express a modified RNA editing protein delivered by an AAV virus and the fact that these ADAR2 like proteins primarily edit only G to A mutations (Sinnamon et al., 2017, 2020).

Despite the excitement for *MECP2* gene replacement and RNA editing strategies, other therapeutic methods are in development. One strategy explored by the Zoghbi lab in Rett model mice is deep brain stimulation. Electrode based stimulation of symptomatic *Mecp2^–/+^* female mice normalized learning and memory defects (Hao et al., 2015; Pohodich et al., 2018). Another promising area for potential Rett therapies, is the repurposing of existing FDA approved compounds. For example, in 2020, it was found that the diabetes drug, metformin, reduces mitochondrial defects and damage from oxidative stress in *Mecp2 308^–/+^* truncation female mice (Zuliani et al., 2020). Finally, a recently developed compound for Alzheimer’s disease reduced motor defects, learning deficits and breathing abnormalities in *Mecp2^–/+^* female mice with minimal side effects (Kaufmann et al., 2019). Until effective and safe *MECP2* gene replacement and/or RNA editing strategies are available, existing or novel chemical compounds could be used to reduce the most severe symptoms in Rett patients.

## Discussion

Currently, there is debate as to whether patients with overlapping phenotypes have Rett syndrome or some other disease. For example, patients with mutations in *CDKL5* were considered to have a severe variant of Rett, however, this was later categorized as a separate disease (Fehr et al., 2013). Patients with *FOXG1* mutations are currently classified with Rett. However, as these rare *FOXG1* mutation patients present with disease from birth and have other symptoms that do not overlap with Rett, a proposal to classify these patients as a separate syndrome was recently submitted (Cutri-French et al., 2020). Therefore, with few exceptions, any discussion about Rett syndrome phenotypes is about how defective *MECP2/Mecp2* expression presents in patients and animal models and manifests as a neurodevelopmental disease.

At this time it is still unclear how *MECP2* expression defects in non-neural tissues contribute to Rett phenotypes. The answer may be complex as illustrated by a report from the Landsberger lab showing that male Bird *Mecp2* deletion mice have abnormal muscle tissue while deletion of the Bird *Mecp2* allele only in muscle resulted in normal muscle tissue (Conti et al., 2015). The conclusion from this study is that non-cell-autonomous signaling to the muscle by defective neurons in the Bird *Mecp2* deletion mice contributes to the muscle defect (Conti et al., 2015). So, there is the possibility that although *MECP2* transcripts are abundant in all tissues and cell types, extracellular neuronal signaling could be responsible for some, if not all of the defects.

The recent identification of MeCP2 binding beyond methylated CpG sites has greatly expanded the range of potential functions of the protein. These advances have been driven by the discovery of additional nucleotide modifications in mammalian brain. Yet many questions remain. For example, does MeCP2 binding to 5-hmc (Kriaucionis and Heintz, 2009) in cerebellum have a significant neurologic function (Mellén et al., 2012)? Do MeCP2 isoforms have different 5-hmc binding activity? Some data suggests that MeCP2 protects 5mc from TET1 conversion to 5 hmc (Szulwach et al., 2011) but more study is needed to resolve this important question. The 2014 discovery of DNMT3A dependent CH methylation in mouse brain by the Song lab (Guo et al., 2014) and the description of MeCP2 binding to methyl CpA sites in brain *in vivo* (Gabel et al., 2015) has been suggested to be key to the pathogenesis of Rett (Lavery et al., 2020). Here again additional research is needed to clearly establish the effect of MeCP2 binding outside of methyl CpG sites.

Another evolving area of research is how MeCP2 functions as a transcriptional repressor and activator. The model of MeCP2 as a transcriptional repressor of single copy genes was developed over twenty years ago (Nan et al., 1997; Jones et al., 1998). Since that time it was found that MeCP2 binds to partially CpG methylated promoters of active genes in neurons (Yasui et al., 2007) and can act as a transcriptional activator by recruitment of CREB in neurons (Chahrour et al., 2008). The latest evidence suggests that MeCP2 modulates gene transcription depending on co-factor interaction. Future gene expression analyses should focus on the differential activity of the MeCP2-e1 and MeCP2-e2 isoforms.

The role of glial cell defects in Rett syndrome pathology is still being described. However, the very fact that glial cells express *MECP2/Mecp2* and are defective in Rett patients and *Mecp2* null and deficient mice is relevant to neuronal disease phenotypes. As Rett females and *Mecp2^–/+^* deficient heterozygous female mice are mosaic for normal and mutant cells, non-cell autonomous effects are clearly contributing to neuronal dysfunction (Johnson et al., 2017). Analysis of neurons from mosaic Bird *Mecp2^–/+^* deficient female mouse brain and human Rett brain suggest that MeCP2 preferentially represses methylated long genes, Sugino et al. (2014), in a cell autonomous manner in neurons, although non-cell autonomous effects on short genes cannot be excluded (Renthal et al., 2018). As recent study concluded that MeCP2 bias toward long gene regulation may be an artifact of PCR based transcript detection (Raman et al., 2018), single cell analyses using alternatives to current RNA-seq methods in brain cell types will be necessary to resolve the role of non-cell autonomous effects in brains mosaic for wild-type and *Mecp2* mutant neurons and glia.

Although it is widely assumed that Rett patients are phenotypically normal at birth this conclusion is based on limited behavioral observation. As there is little incentive and ethical limitations preclude studying apparently normal human infants, a full developmental time course of the disease is needed in Rett model *Mecp2^–/+^* heterozygous mice that more accurately model Rett *MECP2* mutations such *Mecp2-e1* mutant mice (Yasui et al., 2014). Surprisingly, apart from a study that revealed defects in Bird *Mecp2^–/y^* null male embryonic cells, little analysis has been done of early developmental time points (Bedogni et al., 2016). Clearly, a developmental time analyses of both *Mecp2^–/+^* females and *Mecp2^–/y^* males at embryonic, pre-disease, early disease, and late disease is needed to identify pathological mechanisms.

There are aspects of MeCP2 function that are oddly neglected. For example, surprisingly, few studies have directly examined the ability of MeCP2 to bind to RNA. The studies that do show a strong *in vitro* interaction of MeCP2 with mRNA and siRNA via the RG domain between the MBD and TRD (Jeffery and Nakielny, 2004) and *in vivo* interaction with miRNAs (Khan et al., 2017). A logical progression of these studies would be to examine RNA binding ability of common Rett mutant MeCP2 proteins such as T158M *in vitro* and *in vivo*. The Rett field is lacking studies using more unbiased high-through methods such as targets of RNA-binding proteins identified by editing (TRIBE) (McMahon et al., 2016) which fuses MeCP2 to ADAR, an enzyme that modifies RNA where MeCP2 binds. An additional advantage of this approach is that it can be used for *in vivo* studies.

Finally, unexpected discoveries continue to revitalize the study of MeCP2. Recent cell biology studies reveal that compartmentalization of the nucleus by liquid-liquid phase separation forms critical regulatory compartments such as the nucleolus and heterochromatin (reviewed in Alberti et al., 2019). Now it appears that MeCP2 plays a key role in the formation of nuclear heterochromatin domains according to new *in vitro* studies (Fan et al., 2020; Wang et al., 2020) and that the MeCP2 intrinsically disorder region plays a key role in this process (Li et al., 2020). These results build on the extensive study of how MeCP2 organizes chromatin in neuronal cells and may provide key insights into Rett syndrome defects.

The most interesting advances in MeCP2 research concern Rett therapies, as restoration of MeCP2 in mouse brain led to disease reversal in a mouse model (Guy et al., 2007). Current *MECP2* gene therapy for Rett patients has great potential and some risks. The latest gene therapy treatment of Bird *Mecp2^–/+^* heterozygous mice with AAV9/*MECP2* was able to normalize breathing but high viral doses produced severe liver toxicity and death in some animals (Matagne et al., 2020). While RNA editing strategies for repairing mutant MECP2 have great potential as a therapy, delivery of editing proteins to the brain is also as problematic as delivering functional MeCP2. Therefore, considerable time and effort will be needed to refine *MECP2* gene replacement and RNA editing based therapies. Until then, alternative treatments are needed to treat Rett.

One potential therapy for Rett is deep brain stimulation (DSB) which has been approved by the FDA for epilepsy. Nonetheless, DSB also has its risks as electrodes are implanted into the brain. Existing drugs approved by the FDA for other diseases are being re-directed to treat Rett symptoms. Clinical trials of IGF-1 and IGF-1 analogs that have been approved for short stature are ongoing for Rett and offer some hope of reducing symptoms (Khwaja et al., 2014). Another compound, ANAVEX 2-73 which targets the sigma-1 receptor that affects learning, memory, and neuronal development and is in phase II clinical trials for Alzheimer’s disease is also scheduled for Phase I trials in Rett girls (Kaufmann et al., 2019). In some cases, effective treatment for a specific disease have been employed without fully understanding the mechanisms of the disease process. While this may be the case with Rett syndrome, that should not preclude the continuing research on the evolving molecular function of MeCP2 in neurons and other cell types.

## Author Contributions

DY and OS conceived of, planned and wrote the manuscript. Both authors contributed to the article and approved the submitted version.

## Conflict of Interest

The authors declare that the research was conducted in the absence of any commercial or financial relationships that could be construed as a potential conflict of interest.
